# Pneumonia with Empyemia

**Published:** 1887-07

**Authors:** Henry L. Elsner

**Affiliations:** Syracuse, N. Y.


### *PNEUMONIA WITH EMPYEMIA.*

### RADICAL OPERATION—RECOVERY.

Mrs. H., aet. 65 years, previously healthy, was taken ill on Friday, March 11, 1887. She had had repeated slight chills with a final severe chill, nausea, retching and a short, hacking cough with a moderate amount of expectoration. There was some pain in right side over hepatic dullness and an occasional stitch over the lower part of right thorax posteriorly. A physician who was called diagnosed pneumonia. When I saw her on Monday, the 14th of March, the expectoration had become rusty, the pain in the side somewhat relieved, though still acute, her general condition so alarming that the attending physician had given an absolutely bad prognosis, and the family was momentarily expecting death to relieve her sufferings, without making any effort to avert the expected end. Her pupils were dilated, face cyanosed, extremities cold, surface covered with a cold perspiration, abdomen distended and tender, tongue dry, with a brown streak extending backward through its center. The pulse (to which I wish to call your attention most particularly) was irregular, intermittent, and at times dicrotic, averaging 130 beats per minute; R. 36; T. 101.

*Physical Examination. Inspection*—The right thorax in its lower segment was somewhat larger than the left. The sixth and seventh intercostal spaces were more convex than normal, while the respiratory movement was somewhat limited on the right side. Vocal fremitus was abolished over the lower segment of the right lung for about two and one-half inches upward from base; beyond this for one and one-half inches the vocal fremitus was normal, and over a patch as large as a small fist above vocal fremitus was increased. *Percussion* gave absolute flatness at the base of the lung over a space corresponding to the abolished vocal fremitus, above this normal resonance and over the space of increased fremitus absolute dullness. *Auscultation* gave abolished breathing over the lower part of the right lung, absence of voice sound, above this œgophony, and over the dull patch above there was bronchial breathing, crepitant and subcrepitant rales, a number of larger rales with increased voice sound. Physical signs, therefore, clearly showed that there was slight effusion into the right pleural cavity to a level corresponding to the œgophony; that a space between this and the dullness above was occupied by normal lung tissue, and that the dullness above, with the other physical signs, proved conclusively that we had pneumonic infiltration. The hyperdermic needle proved this conclusion to be correct. The fluid withdrawn was characteristic of ordinary serous effusion. The alarming condition of the patient, with the failing pulse, gave indications for heroic treatment. The patient was given 10 M. of tincture of digitalis, hypodermically, and then repeated by the mouth every hour until the pulse became more tense, regular and less frequent. To this was added from one to two tablespoonfuls of whisky, the patient and pulse being carefully watched for the physiological effects of digitalis. The next day the patient had an irregular and intermittent pulse, though of a better character — 120 per minute; was delirious; had herpes vesicles upon the lower left eyelid and upper lip; T. 100; R.,

36. The digitalis was continued at longer intervals; flax-seed poultices with mustard placed over thorax. By Wednesday, the third day of my attendance, the pulse was regular—95 per minute; T. 100; R. 20. Physical signs the same; sputa rusty; cough painful. The digitalis was discontinued, nytro-glycerine and carbonate of ammonium being substituted.

March 17th—T. 99 4-10, P. 95, R. 30. Physical signs unchanged; less delirious; urine examined with negative result, save diminution of chlorides; sputa still rusty.

March 18th—T. 99, P. 100, R. 20. No resolution in pneumonic patch; bronchial breathing with few rales.

From Sunday, March 20th, until the following Saturday, the temperature remained normal, R. from 23 to 30 per minute, pulse from 80 to 90; but there was a decided increase in the physical signs, denoting effusion into the pleural cavity, with accompanying dyspnœa. It now appeared to me that the patient had progressed sufficiently to allow surgical interference for the relief of the pleural effusion, the pneumonic symptoms having entirely disappeared. You will have noticed in following this history, that the temperature never reached beyond 101; that after the first day of my attendance it never reached beyond 100, and that the average was not more than 99°. At the first examination of the patient, the hypodermic withdrew a clear serum; therefore, I expected, with such a train of symptoms as I have given, more particularly with the low temperature, *almost absence of fever*, to withdraw a clear serous fluid by means of the aspirator.

On Sunday, March 27th, assisted by Dr. Totman, I aspirated, and withdrew *two pounds of pus* from the right pleural cavity. We were prepared for serum, but were surprised to find the effusion purulent. The pus was so thick that it flowed slowly and with difficulty through the cannula. The temperature was 99, R. 36, P. 96, dyspnœa increasing. The aspiration was not beneficial, the flatness increased, and with increasing symptoms of lung compression, I decided to do the radical operation for empyema.

Wednesday, March 30th, under strict antiseptic precautions, without spray, assisted by Dr. Totman, I made a free incision in the seventh intercostal space, about one inch long, the center of the incision forming a right angle with a line drawn from the angle of the scapula downward. This was done without ether, after thoroughly freezing the part. A drainage tube was introduced, and about one quart of pus escaped. We did not hear air enter through the incision after the operation, though it found entrance into the thorax through the tube during the second dressing. The patient bore the operation heroically, and never had a temperature above 99 after the operation. There is nothing noteworthy in the subsequent history, for the patient continued to improve, and is now able to sit up and walk about the house. The tube was made shorter at each dressing, and on the 18th of April it could no longer be introduced. Though the lung is not yet fully expanded, the percussion note is improving, and more air is entering the once consolidated and compressed lung The medical treatment, after the aspiration, consisted in the use of tonics, with nutritious diet, and the best ventilation the surroundings afforded. Without the presence of a specific infection, there can be no empyemia. To this, most modern authorities subscribe the germ of empyemia, and the infecting micrococcus of pneumonia are distinct.

This case was originally one of pleuro-pneumonia; later the germ of empyema was superadded. We conclude, therefore, from this case and its history: 1st. That two specific and different germs can attack a patient at or nearly the same time and produce two different diseases, or change the complexion of the already existing disease.

2d. That there can be empyemia without elevation of temperature, and that the amount of pus may constantly increase without other than pressure symptoms.

3d. That the original pleuro-pneumonia may have been so modified by the germ of empyemia as to prevent the usual high temperature; for it is now well established that poisons in the blood, such as urea in cases of pneumonia, occasionally preclude the usual high temperature, and that such cases pass through the various stages without elevation of body heat.

4th. If pneumonia is complicated with empyemia, early evacuation is indicated certainly as soon as the pneumonic symptoms are relieved.

5th. If life is threatened, it is injudicious to wait for the disappearance of the pneumonia before doing the radical operation.

6th. That the radical operation is indicated after a single aspiration, if the symptoms are not relieved, as the physical signs continue or increase.

7th. That the radical operation is always indicated in adults unless the purulent effusion be small.

8th. That washing out the pleural cavity is not necessary unless there are symptoms of constitutional sepsis, showing themselves by fever, delirium and other symptoms.

9th. That in heart failure from pneumonia, digitalis or other heart tonics must be given to their physiological limit, the patient being carefully watched by the physician or a trained nurse.

